# RETRACTION: Activation of MEK1/2/Nrf‐2 Signaling Pathway by Epstein‐Barr Virus‐Latent Membrane Protein 1 Enhances Autophagy and Cisplatin Resistance in T‐Cell Lymphoma

**DOI:** 10.1155/ancp/9846503

**Published:** 2025-10-29

**Authors:** Analytical Cellular Pathology

RETRACTION: X. Jia, Q. He, M. Zeng, Y. Chen, and Y. Liu, “Activation of MEK1/2/Nrf‐2 Signaling Pathway by Epstein‐Barr Virus‐Latent Membrane Protein 1 Enhances Autophagy and Cisplatin Resistance in T‐Cell Lymphoma,” *Analytical Cellular Pathology* 2021 (2021): 6668947, https://doi.org/10.1155/2021/6668947.

The above article, published online on 19 June 2021 in Wiley Online Library (wileyonlinelibrary.com), has been retracted by agreement between the journal Editor‐in‐Chief, Dimitrios Karamichos; and John Wiley & Sons Ltd.

The retraction has been agreed following an investigation of the concerns raised by *Hoya camphorifolia* on PubPeer [1], which identified multiple instances of figure duplication.

Specifically:

Figure 3c: The KHYG‐1 cells cultured with LMP1+ DMSO are similar to the image of the DU145 cells in Figure 3c (bottom left panel) of [2].

Figure 5d: The SNK‐6 cells treated with shLMP1 +cheirolin appear identical to the Huh‐7 cells cultured with H2O2 shown in Figure 1c of [3].

As a result of the investigation, the data and conclusions of this article are considered unreliable.

The authors have been informed of this retraction but did not provide a response.
